# A case report of giant pancreatic pseudocyst following acute pancreatitis: experience with endoscopic internal drainage

**DOI:** 10.1186/s13104-018-3375-9

**Published:** 2018-04-27

**Authors:** W. A. E. Udeshika, H. M. M. T. B. Herath, S. U. B. Dassanayake, S. P. Pahalagamage, Aruna Kulatunga

**Affiliations:** National Hospital, Colombo, Sri Lanka

**Keywords:** Giant pancreatic pseudocyst, Endoscopic internal drainage, Acute pancreatitis

## Abstract

**Background:**

Pancreatic cysts are being diagnosed more frequently because of the increasing usage of imaging techniques. A pseudocyst with the major diameter of 10 cm is termed as a giant cyst. Asymptomatic pseudo-cysts up to 6 cm in diameter can be safely observed and monitored without intervention, but larger and symptomatic pseudocysts require intervention.

**Case presentation:**

A 27-year-old Sri Lankan male, with history of heavy alcohol use, presented with progressive abdominal distension following an episode of acute pancreatitis. Contrast enhanced CT scan of the abdomen showed a large multilocular cystic lesion almost occupying the entire abdominal cavity and displacing the liver medially and the right dome of the diaphragm superiorly. The largest locule in the right side measured as 30 cm × 15 cm × 14 cm. Endoscopic ultrasound guided drainage of the cyst was performed. The cyst was entered into with an electrocautery-assisted cystotome and a lumen-opposing metal stent was deployed under fluoroscopic vision followed by dilatation with a 10 mm controlled radial expansion balloon. Repeat endoscopic ultrasound was done a week later due to persistence of the collection and a second stent was inserted. Then 10 French gauge × 10 cm double ended pigtails were inserted through both stents. The cysts were not visualized on subsequent Ultra sound scans. Stent removal was done after 3 weeks, leaving the pigtails insitu. The patient made an uneventful recovery.

**Conclusion:**

Giant pancreatic pseudocysts are rare and earlier drainage is recommended before clinical deterioration. Some experts suggest that cystogastrostomy may not be appropriate for the treatment of giant pancreatic pseudocysts and in some instances external drainage of giant pancreatic pseudocysts may be safer than cystogastrostomy. Video-assisted pancreatic necrosectomy with internal drainage and laparoscopic cystogastrostomy were also tried with a good outcome. With our experience we suggest endoscopic guided internal drainage as a possible initial method of management of a giant pseudo cyst. However long-term follow up is needed with repeated imaging and endoscopy. In instances where the primary endoscopic internal drainage fails, surgical procedures may be required as a second line option.

## Background

Pancreatic cysts are being diagnosed more frequently because of the increasing usage of imaging techniques. 15–30% of these cysts are pancreatic pseudocysts [1] and occurs in approximately 10–20% of cases of acute pancreatitis [2]. They can be single or multiple and present with a wide range of clinical manifestations depending upon the location, size and the presence of infection. A pseudocyst with the major diameter of 10 cm is termed as a giant cyst [3, 4] and now infrequently seen due to modern diagnostic and therapeutic methods. The diagnosis of a pancreatic pseudocyst needs imaging with ultra-sonography, CT scan or MRI. Asymptomatic pseudo-cysts up to 6 cm in diameter can be safely observed and monitored with serial imaging but larger and symptomatic pseudocysts require intervention [5]. Here we describe a young male who developed multiple giant pseudocysts with a very large cyst compressing intra-abdominal organs following acute pancreatitis. Only very few cases of very giant pancreatic pseudocysts were found on literature review.

## Case presentation

A 27-year-old Sri Lankan male was admitted to a local hospital with severe epigastric pain and vomiting following a binge of alcohol, 2 months prior to admission to our unit. He was taking three units of alcohol daily during last 6 years. The abdomen was distended with free fluid. Examination of the other systems was normal. His serum amylase was significantly elevated on admission and CRP level after 48 h was elevated (Table 1).

**Table 1:** Heamatological and biochemical investigations of the first admission

Amylase = 6267 U/L	ALT = 22 U/L	AST = 34 U/L
WBC = 9 × 10^9^/L	Hb = 10.6 g/dL	Platelet = 506 × 10^9^/L
Na+ = 134 mmol/L	K+ = 4.1 mmol/L	Serum creatinine = 83 µmol/L
T.bilirubin = 14 µmol/L	Albumin = 26 g/L	Globulin = 22 g/L
INR = 1.4	FBS = 3.8 mmol/L	
CRP after 48 h = 295 mg/L	ESR = 50 in 1st hour	Ca^2+^ = 1.25 mmol/L

Chest X-ray revealed a small pleural effusion on the right side. Ultra sound scan of the abdomen showed a coarse echogenic liver with gross ascites.

Following this episode he had persistent anorexia and weight loss with intermittent abdominal pain. He did not have fever or vomiting. Bowel opening was normal. He was subsequently transferred to our unit from the local hospital with worsening abdominal pain and distension. On clinical examination he was emaciated and was mildly pale. He was not icteric. He was hemodynamically stable and had a right sided small pleural effusion. Abdomen was distended and tense. Investigations revealed high amylase and low albumin (Table 2).

**Table 2:** Heamatological and biochemical investigations of the second admission

Amylase = 1524 U/L	ALT = 23 U/L	AST = 34 U/L
WBC = 10.57 × 10 9/L	Hb = 9.9 g/dL	Platelet = 352 × 10^3^/L
Na+ = 134 mmol/L	K+ = 3.9 mmol/L	Serum Creatinine = 76 µmol/L
INR = 1.3	FBS = 4.3 mmol/L	
T.bilirubin = 7.3 µmol/L	Albumin = 22 g/L	Globulin = 27 g/L
CRP after 48 h = 72 mg/L	ESR = 58	Ca^2+^ = 1.13 mmol/L

**Fig. 1:**
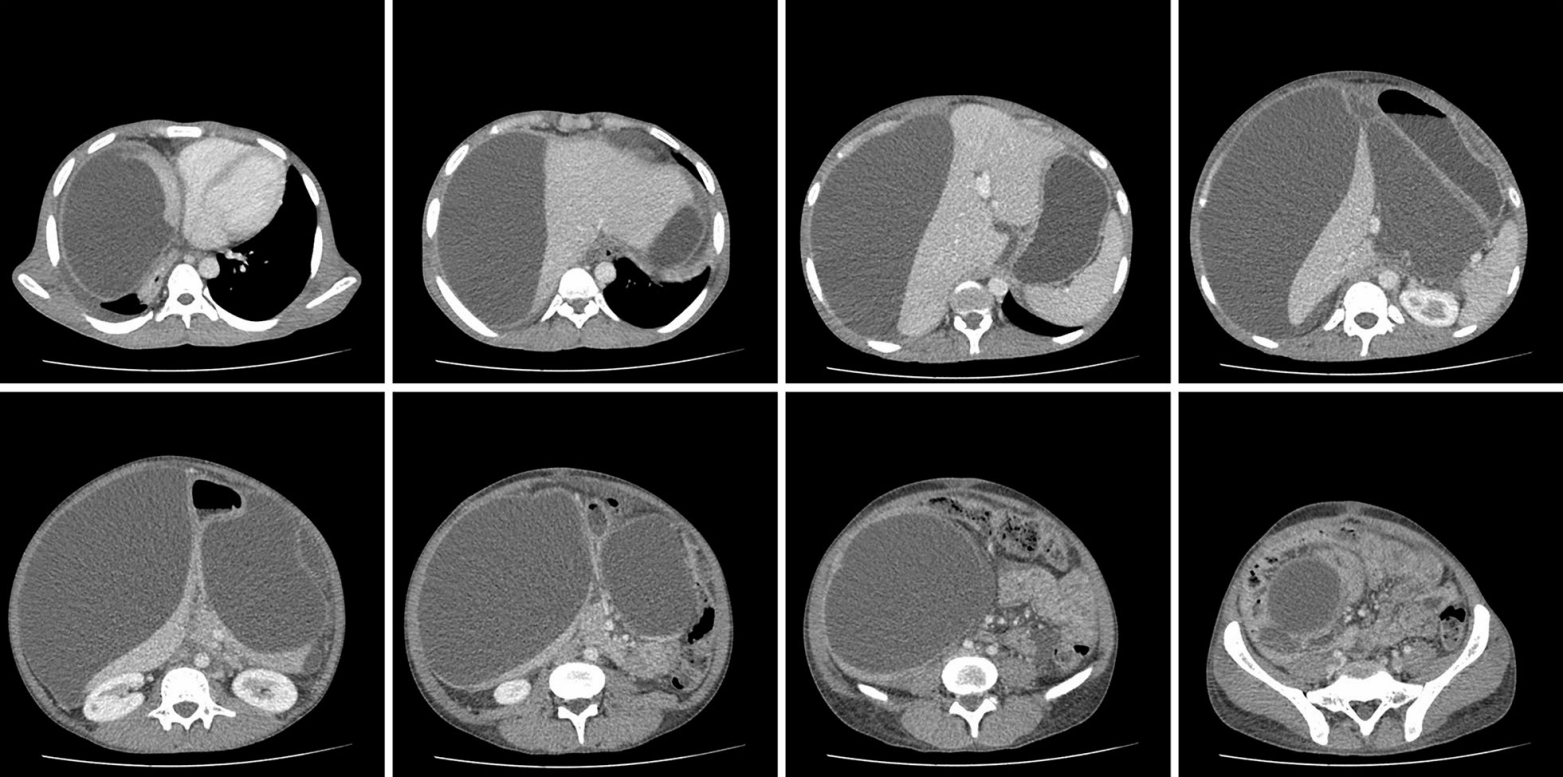
Contrast enhanced CT scan of the abdomen—axial view—large multilocular cystic lesion occupying almost the entire abdominal cavity

**Fig. 2:**
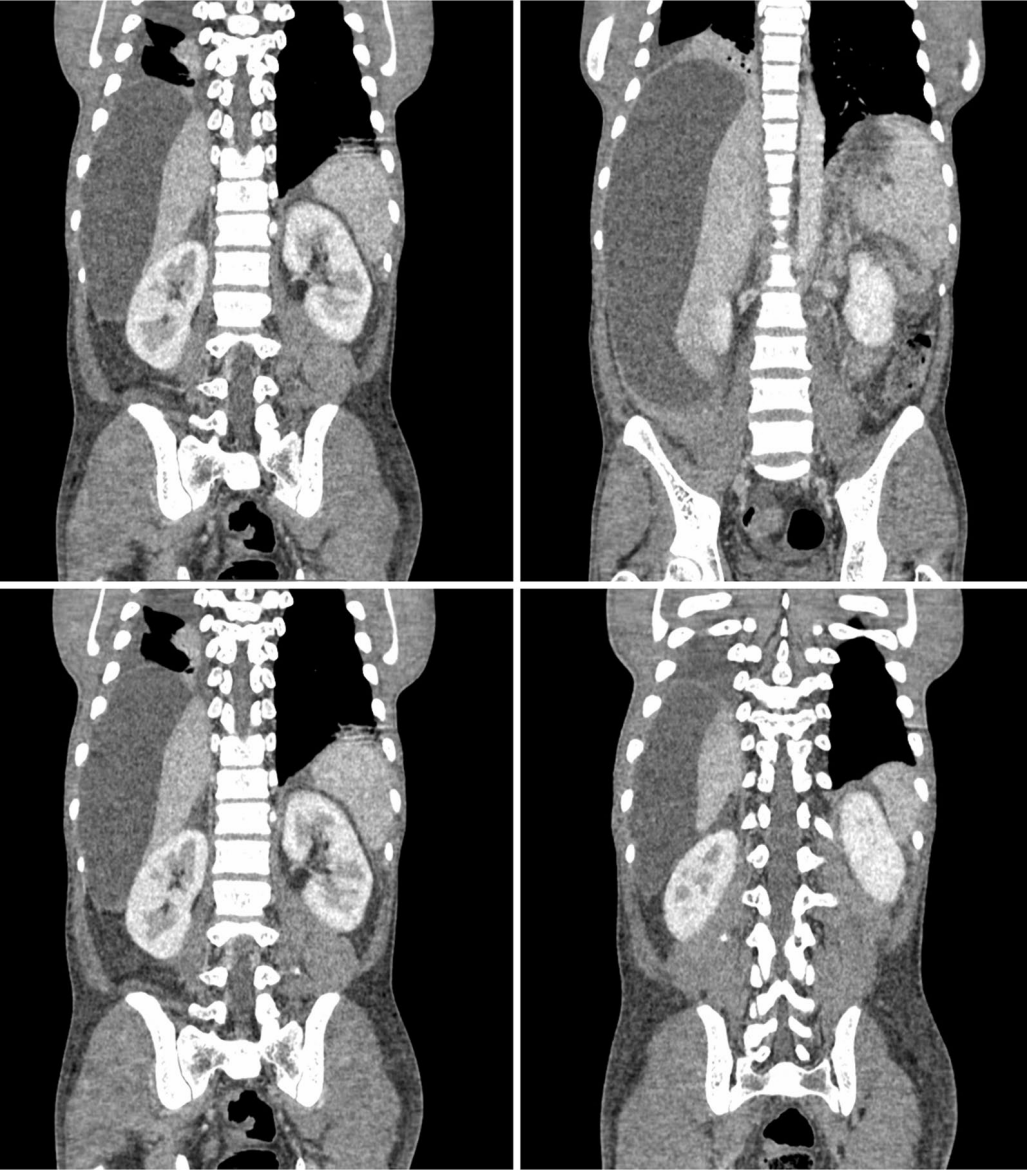
Contrast enhanced CT scan of the abdomen—coronal view

**Fig. 3:**
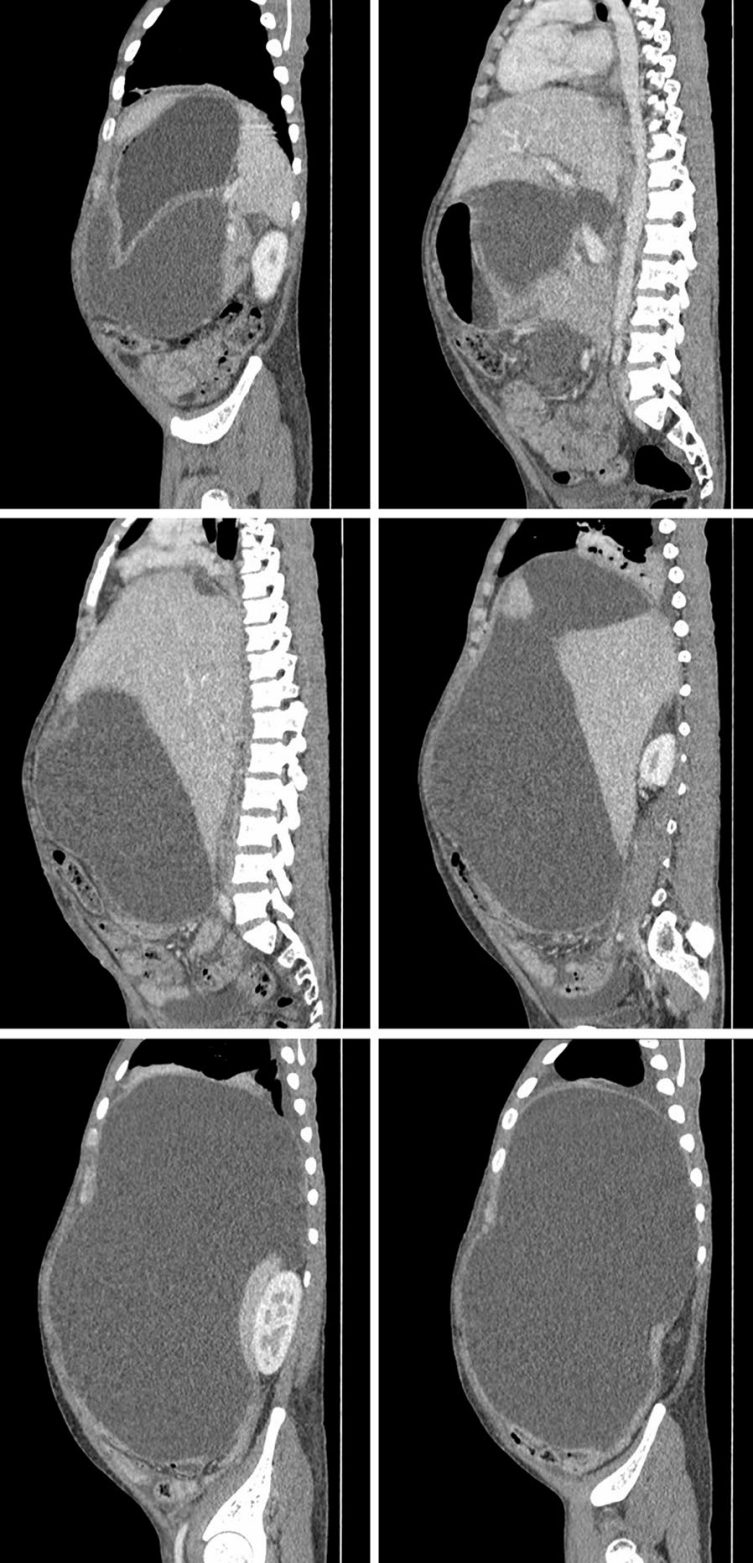
Contrast enhanced CT scan of the abdomen—sagittal view

Contrast enhanced CT scan of the chest and abdomen showed a large multilocular cystic lesion occupying almost the entire abdominal cavity. The largest locule in the right side measured 30 cm × 15 cm × 14 cm displacing the liver medially and the right dome of the diaphragm superiorly. Two smaller locules were seen in the lesser sac, compressing the central abdominal structures. Cysts contained clear fluid with no enhancement of the walls. No separations or evidence of haemorrhage was seen (Figs. 1, 2, 3). There was a small right side pleural effusion with collapse consolidation of the medial segment of the right lower lobe. Pancreas appeared as a thin line likely to be due to partial atrophy and compression and there were no duct dilatations, calcifications or necrosis. A small locule of fluid was seen in relation to the tail of the pancreas. Heart was deviated to the left, due to the largest cyst which was extending into the right hemi thorax. No Para aortic or pelvic lymph node enlargement was seen. The liver, gallbladder and spleen appeared normal. Both kidneys were normal in size with normal contrast enhancement. There was a small amount of free fluid in the pelvis.

**Fig. 4:**
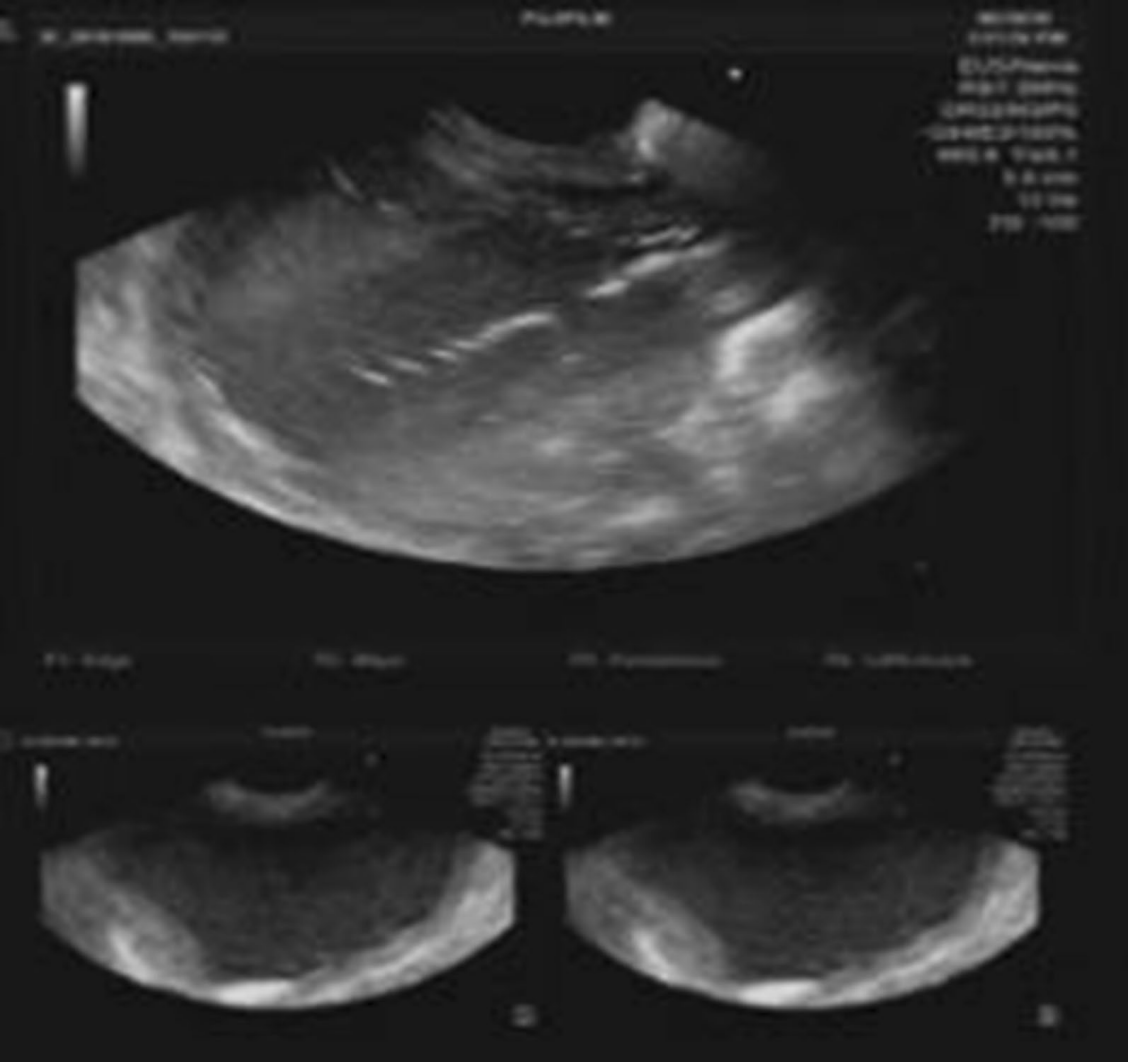
EUS image showing cystotome inside cyst cavity (above) and images of the cyst before puncture (below)

**Fig. 5:**
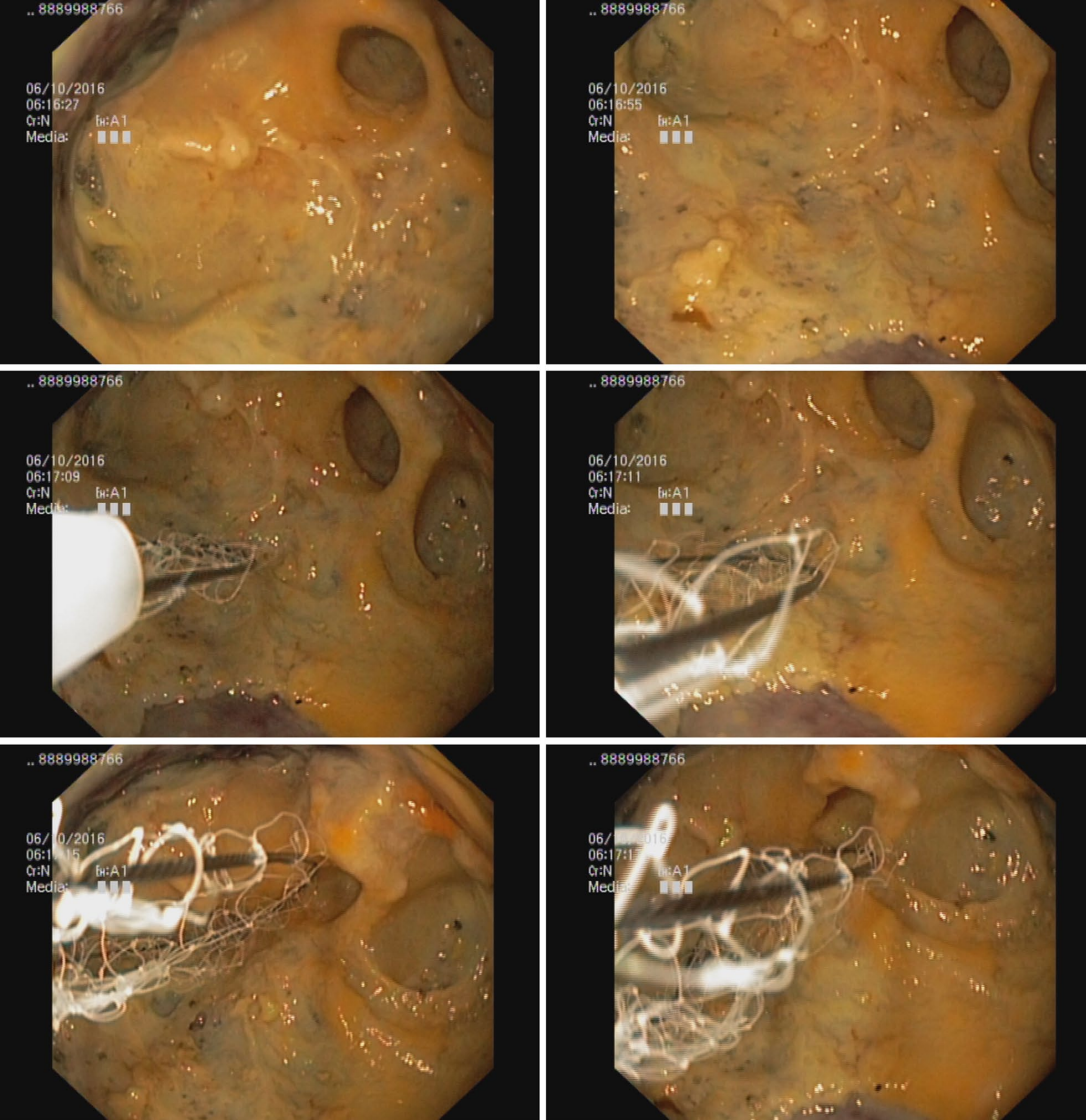
Endoscopic view during cyst debridement

**Fig. 6:**
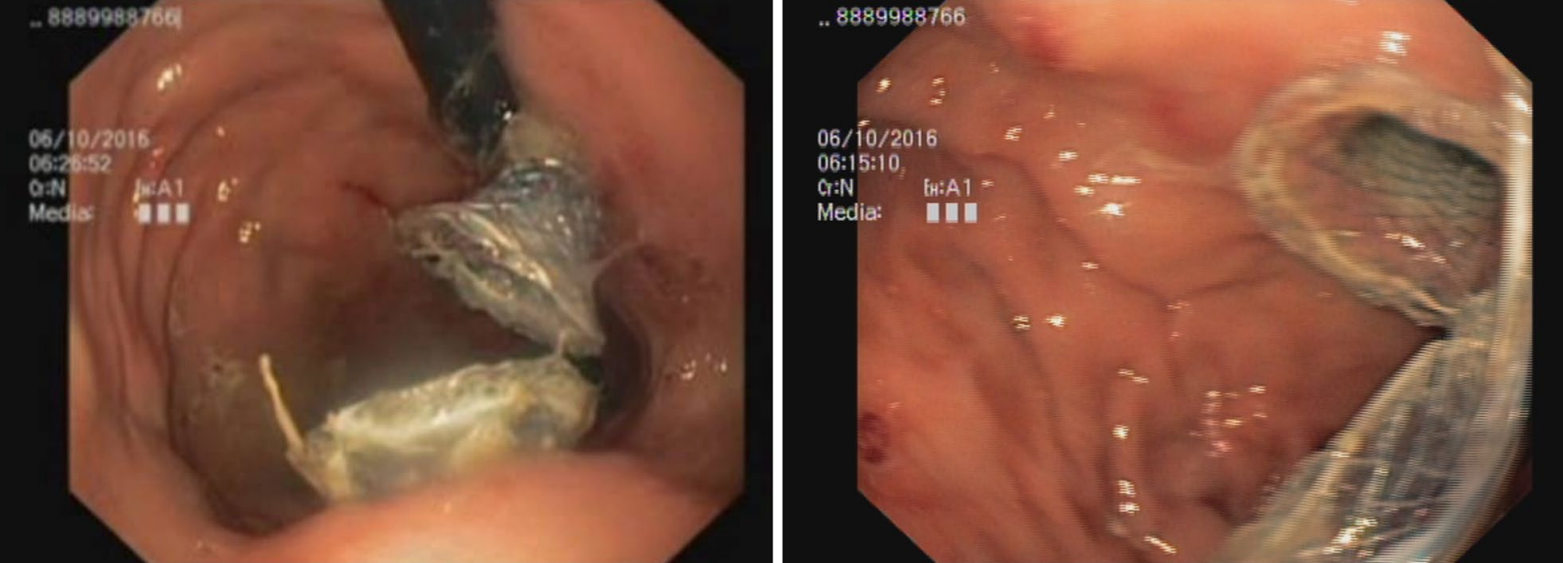
Retroflexed view showing two LEMS insitu

Endoscopic ultrasound (EUS) guided drainage was planned as a therapeutic procedure and was performed using a large channel linear-array echoendoscope (Fujinon, Tokyo, Japan). On the EUS, the pseudocyst was visualized bulging into the posterior-lateral wall of the stomach. The cyst was entered into with an electro cautery-assisted cystotome (Wilson Cook, Winston Salem, NC, USA) and a 0.035 in. guide wire was introduced (Fig. 4). A 15 mm × 30 mm lumen-opposing metal stent(LEMS) (Boston Scientific, Marlborough, MA, USA) was deployed under fluoroscopic vision. Dilatation was done with a 10 mm CRE balloon. A large amount of clear fluid was drained (Fig. 5). Repeat EUS was done a week later due to persistence of the collection on conventional ultra sound scan. A loculated portion of the cyst was seen to be persisting and a second 15 mm × 20 mm LEMS was inserted by an identical procedure (Fig. 6). Pus discharge was noted from the previous stent, which was cleaned and washed out. Then 10Fr × 10 cm double ended pigtails were inserted into both stents. The peri-procedure period was covered with broad-spectrum antibiotics. The cysts were not visualized on subsequent ultra sound scans. Stent removal was done after 3 weeks leaving the pigtails insitu. The patient made an uneventful recovery and was discharged to be reviewed in 6 weeks.

## Discussion and conclusions

According to the Atlanta Symposium, a fluid collection more than 4 weeks old and surrounded by a wall is defined as a pancreatic pseudocyst [6]. In acute pancreatitis, necrosis of peripancreatic tissue or parenchyma can cause liquefaction and subsequent organization resulting in pseudocysts. In chronic pancreatitis, an acute exacerbation of pancreatitis or progressive ductal obstruction can cause pseudocyst formation. Also a blunt or penetrating trauma and injury during pancreatic surgeries can disrupt the pancreatic duct causing pseudocyst formation. These cysts can present with a wide range of clinical manifestations such as abdominal pain, duodenal or biliary obstruction, vascular occlusion, fistula formation into pleural space or pericardial space and digestion of an adjacent vessel resulting in pseudoaneurysms. The diagnosis of pancreatic pseudocysts is made using imaging techniques in the appropriate clinical context.

Giant acute pseudo-pancreatic cyst can occur after acute pancreatitis and they measure 10 cm or more in major diameter. Several giant cysts have been reported in literature. Bozeman in 1882 reported the largest pancreatic pseudocyst cyst, which weighed 10 kg [7]. Other reported cases are a giant pseudocyst containing about 6100 mL of fluid [8], one measuring 25 × 17 cm, containing 4.5 L of fluid [7], 25.7 cm × 15.3 cm × 10.9 cm sized one containing 3 L of [9], one with a diameter of 21 cm [10] and another one with diameter of 22 cm [11]. The pseudocyst we describe here is much larger than these pseudocysts and its largest locule measured 30 cm × 15 cm × 14 cm in size.

In one study of 74 patients with pseudocysts following acute pancreatitis, those with a high Ranson score were at a significant risk for developing giant pseudocysts and worse outcome [12]. Our patient did not have any organ failure during the first episode of acute pancreatitis but the 48-h CRP was high. We were unable to calculate Ranson score.

The management of pseudocysts is based on the studies done in the past that showed pseudocysts persisting beyond 6 weeks rarely resolve and develop complications nearly in about 50% during continued observation. Bradley EL and colleagues concluded that prolonged observation of pancreatic pseudocysts beyond 7 weeks greatly exceeded the mortality of elective surgery [13]. According to Shatney optimal timing of the operation in patients with uncomplicated pseudocysts appears to be around 4 weeks [14]. Vitas suggested a more conservative approach concluding that nonoperative, noninterventional, expectant approach is warranted in the management of selected patients with pancreatic pseudocysts [5]. In 1990 Yeo and colleagues followed up 75 patients with pancreatic pseudocysts and showed that a large proportion of patients with pancreatic pseudocysts, without specific indications for operative treatment, can be safely managed non-operatively with careful clinical and roentgenographic follow-up study [15]. In this study Pseudocysts greater than 6 cm in diameter required surgical treatment significantly more frequently compared to pseudocysts of less than 6 cm in diameter [15]. The patient in our case required interventions due to the pseudocyst being large and symptomatic.

Radiologic imaging with percutaneous catheter drainage and endoscopic drainage are now two additional treatment options. Surgical drainage was the only form of therapy in the past comprising of internal drainage (in the form of a cystogastrostomy, cystoduodenostomy [16] or a Roux-en-Y- cystojejunostomy), external drainage or excision of the cyst. In a recent randomized trial in 2013, comparing efficacy of endoscopic and surgical cystogastrostomy for pancreatic pseudocyst drainage, both methods were to be of similar efficacy. However, endoscopic treatment was associated with shorter hospital stays and better physical and mental health of patients with a lower cost [17]. In another two studies endoscopic drainage of pancreatic-fluid collections was successful in the majority of patients and was highly effective [18, 19]. The addition of endoscopic ultrasonography for endoscopic drainage is a new development making it minimally invasive, effective and a safe approach with reduce risk associated with endoscopic drainage [20, 21]. A study that compared endoscopic and percutaneous drainage of symptomatic pancreatic fluid collections concluded that endoscopic drainage was associated with higher rates of treatment success, lower rates of re-intervention and shorter lengths of hospital stay [22].

There are only few case studies in literature regarding the management of giant pseudo cysts. Behrman and colleagues concluded that expectant management of giant pseudo cysts was associated with higher morbidity and mortality than with small pseudo cysts. They suggested that earlier external drainage, before clinical deterioration, may be beneficial in giant pseudocysts [12]. Wang and colleagues performed an open cystogastrostomy on a 65-year-old man with a giant pancreatic pseudocyst and he recovered uneventfully [9]. Johnson and colleagues reported four patients with giant pseudocysts treated by cystogastrostomy who developed postoperative complications as a result of incomplete emptying of the cyst. From this study they concluded that cystogastrostomy might not be appropriate for the treatment of giant pancreatic pseudocysts as it failed to provide dependent drainage of a large cysts in these patients. It was also concluded that if internal drainage was performed, the cyst should be anastomosed to a defunctional loop of jejunum in a dependent position. They also stated that in some instances external drainage of giant pancreatic pseudocysts may be safer than cystogastrostomy [23]. Ten patients with acute giant pseudocysts underwent video-assisted pancreatic necrosectomy at the time of internal drainage and this was shown to prevent postoperative retroperitoneal complications. This study illustrated that depending on appropriate surgical timing, video-assisted necrosectomy is a feasible and safer procedure when managing giant pseudocysts [3]. Laparoscopic cystogastrostomy was successfully done on a 60-year-old lady with a giant pseudocyst of the pancreas with a good outcome [24]. Here we performed endoscopic ultrasonography and inserted two stents and pigtails to drain the cyst. Later the two stents were removed leaving the pigtails in situ. Patient’s symptoms improved with the drainage of pseudocyst and did not required open surgery. However three repeated endoscopy procedures had to be performed and had a prolong hospital stay of 3 weeks.

With our experience we suggest endoscopic guided internal drainage as a possible initial method of management of giant pseudo cysts. However, long-term follow up is needed to make sure that it does not recur and multiple repeated endoscopy might be needed. In instances, in which the primary endoscopic internal drainage fails, surgical procedures may be required as a second line option.

In conclusion, giant pancreatic pseudocysts are rare and only few case reports are available on its management. Earlier surgical drainage was the only option for pseudocysts but lately radiologic imaging with percutaneous catheter drainage and endoscopic drainage have become available. Earlier drainage was suggested for giant pseudocysts before clinical deterioration. Some suggest that cystogastrostomy may not be appropriate for the treatment of giant pancreatic pseudocysts and in some instances external drainage of giant pancreatic pseudocysts may be safer than cystogastrostomy. Video-assisted pancreatic necrosectomy with internal drainage and laparoscopic cystogastrostomy were also tried with good outcomes. Here we describe a patient with giant pseudocyst following acute pancreatitis who underwent endoscopic ultrasonography and internal drainage of the cyst using stents and pigtails successfully. This can be used as a primary treatment method for giant pseudo cysts although long term follow up with imaging is necessary.

## Abbreviations

CECT: contrast enhanced computed tomography
MRI: magnetic resonant imaging
CRP: C reactive protein
EUS: endoscopic ultra sonography
LEMS: lumen-opposing metal stent

## References

[1] Fasanella KE, McGrath K (2009). Cystic lesions and intraductal neoplasms of the pancreas. Best Pract Res Clin Gastroenterol.

[2] Memis A, Parildar M (2002). Interventional radiological treatment in complications of pancreatitis. Eur J Radiol.

[3] Oria A (2000). Internal drainage of giant acute pseudocysts: the role of video-assisted pancreatic necrosectomy. Arch Surg.

[4] Soliani P (2004). Pancreatic pseudocysts following acute pancreatitis: risk factors influencing therapeutic outcomes. JOP.

[5] Vitas GJ, Sarr MG (1992). Selected management of pancreatic pseudocysts: operative versus expectant management. Surgery.

[6] Bradley EL (1993). A clinically based classification system for acute pancreatitis. Summary of the International Symposium on Acute Pancreatitis, Atlanta, Ga, September 11 through 13, 1992. Arch Surg.

[7] Shah SA (2012). Giant pancreatic pseudocyst. J Coll Physicians Surg Pak.

[8] Walker LG, Stone HH, Apple DG (1967). Pseudocysts of the pancreas: a review of 59 cases. South Med J.

[9] Wang GC, Misra S (2015). A giant pancreatic pseudocyst treated by cystogastrostomy. BMJ Case Rep..

[10] De Socio GV (2012). A giant pancreatic pseudocyst in a patient with HIV infection. J Int Assoc Physicians AIDS Care.

[11] Yamaguchi T (2007). Huge pseudocyst of the pancreas caused by poorly differentiated invasive ductal adenocarcinoma with osteoclast-like giant cells: report of a case. Hepatogastroenterology.

[12] Behrman SW, Melvin WS, Ellison EC (1996). Pancreatic pseudocysts following acute pancreatitis. Am J Surg.

[13] Bradley EL, Clements JL, Gonzalez AC (1979). The natural history of pancreatic pseudocysts: a unified concept of management. Am J Surg.

[14] Shatney CH, Lillehei RC (1981). The timing of surgical treatment of pancreatic pseudocysts. Surg Gynecol Obstet.

[15] Yeo CJ (1990). The natural history of pancreatic pseudocysts documented by computed tomography. Surg Gynecol Obstet.

[16] van Heerden JA, ReMine WH (1975). Pseudocysts of the pancreas. Review of 71 cases. Arch Surg.

[17] Varadarajulu S (2013). Equal efficacy of endoscopic and surgical cystogastrostomy for pancreatic pseudocyst drainage in a randomized trial. Gastroenterology.

[18] Hookey LC (2006). Endoscopic drainage of pancreatic-fluid collections in 116 patients: a comparison of etiologies, drainage techniques, and outcomes. Gastrointest Endosc.

[19] Varadarajulu S (2011). Endoscopic transmural drainage of peripancreatic fluid collections: outcomes and predictors of treatment success in 211 consecutive patients. J Gastrointest Surg.

[20] Lopes CV (2007). Endoscopic-ultrasound-guided endoscopic transmural drainage of pancreatic pseudocysts and abscesses. Scand J Gastroenterol.

[21] Yasuda I (2009). EUS-guided pancreatic pseudocyst drainage. Dig Endosc.

[22] Keane MG (2016). Endoscopic versus percutaneous drainage of symptomatic pancreatic fluid collections: a 14-year experience from a tertiary hepatobiliary centre. Surg Endosc.

[23] Johnson LB, Rattner DW, Warshaw AL (1991). The effect of size of giant pancreatic pseudocysts on the outcome of internal drainage procedures. Surg Gynecol Obstet.

[24] Golash V, Cutress R (2005). Laparaoscopic cytogastrostomy for a giant pseudocyst of pancreas. Surgeon.

